# Active compounds from *Calendula officinalis* flowers act via PI3K and ERK signaling pathways to offer neuroprotective effects against Parkinson's disease

**DOI:** 10.1002/fsn3.3792

**Published:** 2023-10-26

**Authors:** Xuanming Zhang, Rongchun Wang, Nataliya Finiuk, Rostyslav Stoika, Houwen Lin, Xue Wang, Meng Jin

**Affiliations:** ^1^ Engineering Research Center of Zebrafish Models for Human Diseases and Drug Screening Biology Institute, Qilu University of Technology (Shandong Academy of Sciences) Jinan China; ^2^ Department of Regulation of Cell Proliferation and Apoptosis Institute of Cell Biology, National Academy of Sciences of Ukraine Lviv Ukraine; ^3^ Research Center for Marine Drugs, State Key Laboratory of Oncogenes and Related Genes, Department of Pharmacy, School of Medicine Shanghai Jiao Tong University Shanghai China

**Keywords:** *Calendula officinalis*, functional food, LC–MS/MS, neuroprotective effects, Parkinson's disease, signaling pathway

## Abstract

*Calendula officinalis* flowers, associated with diverse biological effects, could be utilized as functional food ingredients to play a crucial role in human health. In this study, we examined the anti‐PD activity of *C. officinalis* flower extracts and investigated their bioactive compounds and molecular mechanisms based on LC–MS/MS assay, bioinformatic exploration and in vitro treatment of SH‐SY5Y cells. *C. officinalis* extracts exhibited significant positive effects on the length and fluorescence density of the dopaminergic neuron region in zebrafish larvae. At 10 μg/mL, the extract restored the length to 96.54% and fluorescence density to 87.77% of the control values, which was equivalent to the effect of a positive drug, indicating the extract's powerful potential to alleviate PD symptoms. Five active compounds, including chlorogenic acid, 3,4‐dicaffeoylquinic acid (DA), rutin, isorhamnetin 3‐O‐glucoside (IG) and calenduloside E (CE) were identified in extracts by LC‐QTOF‐MS/MS. Hsp90α, PI3K and ERK were revealed as core targets of DA, IG and CE in relation to anti‐PD activity. The compounds docked deeply within the pocket region of Hsp90α protein, and their binding energies (∆*G*_b_) were −6.93 kcal/mol (DA), −6.51 kcal/mol (IG) and −3.03 kcal/mol (CE), respectively. Subsequently, they concurrently activated the PI3K/Akt signaling pathway and inhibited the ERK signaling pathway, thereby preventing neuronal death and alleviating neuronal degeneration. These compounds from *C. officinalis* could be potent nutraceutical agents with protective properties that may shield dopaminergic neurons against the damage caused by PD. Our findings provide a basis for utilizing the *C. officinalis* flowers in functional foods.

## Graphical abstract

In this study, we aimed to examine the anti‐PD components of *C. officinalis* flowers and investigate their molecular mechanisms. Five active compounds from the extracts were identified via LC‐QTOF‐MS/MS. 3,4‐Dicaffeoylquinic acid, isorhamnetin 3‐O‐glucoside and calenduloside E were shown to bind to the pocket region of the Hsp90α protein and then concurrently activate the PI3K/Akt signaling pathway and inhibit the ERK signaling pathway, thereby preventing neuronal death and alleviating neuronal degeneration.

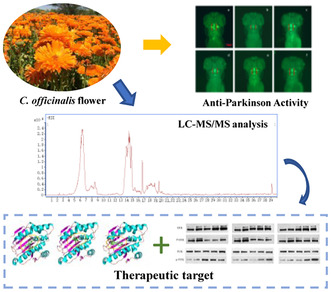

## 1 INTRODUCTION

Parkinson's disease (PD) is one of the most common neurodegenerative disorders (Bloem et al., 2021). As the population ages, by 2030, China is predicted to have the largest number of PD patients in the world. Currently, the underlying mechanisms of PD are not quite clear. Cases are believed to be the result of a complex interaction between genetic predisposition and environmental factors in individuals with many risk factors such as aging, family history, chemical exposure, etc. (Balestrino & Schapira, 2020; Funayama et al., 2023). PD patients experience symptoms of rest tremor, rigidity, bradykinesia and stooping posture, which are associated with progressive loss of dopaminergic neurons in the substantia niagra pars compacta and significantly reduced levels of dopamine in the striatum (Trager et al., 2020; Yang et al., 2020). The causes of neuronal loss in PD include, but are not limited to, mitochondrial dysfunction, oxidative injury and inflammatory changes in the brain, which lead to cellular dysfunction and death through apoptosis or autophagy. PD has profound consequences for patients and has become a subject of intensive research. The development of surgical interventions and innovative agents are crucial to relieving PD symptoms.

Natural herbs, owing to their diversity in chemical composition, are considered to have unique biological activities and drug‐like properties (Della Valle et al., 2020; Mohammed et al., 2020). *Calendula officinalis*, namely Jin‐Zhan‐Ju, has long been used as a folk remedy in Traditional Chinese Medicine. Generally, the leaves, flowers and roots of the plant were reported to possess potential therapeutic efficacy (Ak et al., 2021; Patil et al., 2022). Decoctions of its flowers have been used as posset drinks in the western market. Previous studies have demonstrated the presence of a number classes of chemical compounds in this species (Dhingra et al., 2022; Vitale et al., 2022), including flavonoids, phenolic acids, saponins, carotenoids, sterols, lipids, etc., which have been confirmed to exhibit a broad range of pharmacological effects related to anti‐inflammatory, anti‐HIV, anticancer, immunostimulant, as well as antibacterial and antioxidant, activities (Cruceriu et al., 2020; Tanideh et al., 2020; Tung et al., 2019). In this study, we aimed to examine the anti‐PD components of *C. officinalis* flowers and investigate their molecular mechanisms via bioinformatics analysis and testing SH‐SY5Y cells in vitro. The results of our work provided insights into developing *C. officinalis* extracts as products that protect brain health.

## 2 MATERIALS AND METHODS

### 2.1 Chemicals, reagents and sample preparation

Chlorogenic acid, rutin, 3,4‐dicaffeoylquinic acid (DA), isorhamnetin 3‐O‐glucoside (IG), calenduloside E (CE), curcumin, nomifensine and MPTP (1‐methyl‐4‐phenyl‐1,2,3,6‐tetrahydropyridine) were purchased from Shanghai Yuanye Biotechnology Co. Ltd. MPP (dihydrochloride) was purchased from Sigma‐Aldrich. Acetonitrile and MS‐grade water were acquired from Tedia Company Inc. and Watsons Ltd., respectively. All other chemicals were of analytical grade. SH‐SY5Y cells were obtained from the American Type Culture Collection (ATCC). Fresh flower heads of *C. officinalis* were obtained from the Anguo medicine market, Hebei Province. One gram of plant material was extracted with 8 mL of 70% ethanol for 1 h using ultrasound, and, subsequently, the solution was concentrated to acquire the residue (111.4 mg).

### 2.2 Neuroprotective assay in the zebrafish model

Transgenic Tg (vmat2: GFP) zebrafish were obtained from the Zebrafish Drug Screening Platform at the Biology Institute and maintained on a 14 h light/10 h dark cycle at 28°C. *C. officinalis* extract was evaluated for its neuroprotective effect on zebrafish larvae using an MPTP‐induced PD model. Twenty‐four hours postfertilization, larvae were transferred into six‐well plates at a density of 15 larvae per well. The experiments were divided into several groups: a vehicle control group (embryo medium); a model group (50 μM MPTP); a positive group (50 μM MPTP + 0 μM nomifensine); and intervention groups (50 μM MPTP +2.5, 5 or 10 μg/mL of extract). After treatment, zebrafish were incubated at 28°C continuously for 72 h. Ultimately, the zebrafish were photographed under a fluorescence microscope (AXIO Zoom.V16, Zeiss, Dresden, Germany), and the length and fluorescence density of their DA neuron regions were determined with Image‐Pro Plus 5.1 software for anti‐PD assessment.

### 2.3 LC–MS/MS analysis

Extracts (2 mg/mL) that had been passed through a 0.45 μm filter were subjected to an Agilent LC1200/MS Q‐TOF6520 system. LC–MS was performed with solvents A (water) and B (acetonitrile) on an XDB‐C_18_ HPLC column (4.6 × 250 mm, 5 μm; Agilent). The gradient conditions were as follows: 5%–20% B (0–10 min), 20%–80% B (10–35 min) and 80%–100% B (35–40 min). The following MS conditions were employed: scanning range of 100–1500 m/z in ESI negative mode, nebulizer pressure of 45 psi, drying gas temperature of 350°C, drying gas flow of 10 L/min and capillary voltage of 4000 V. Compounds were identified by high‐resolution tandem mass spectrometry (HR‐MS/MS) and comparison of the retention times with those of standards.

### 2.4 Exploration of targets using network pharmacology

The molecular structures of DA, IG and CE were acquired from the PubChem database (http://pubchem.ncbi.nlm.nih.gov/) for network pharmacology analysis. The potential targets of the components were screened using the Swiss Target Prediction Database (http://www.swisstargetprediction.ch/). The PD target information was collected using the GeneCards (https://www.genecards.org/) and DisGeNET (http://www.disgenet.org/) databases with the search term “Parkinson's disease”. Information on protein interactions was acquired by inputting the candidate targets to String 11.5 (https://string‐db.org/). Furthermore, the PD therapeutic mechanism was investigated according to a compound‐target (C‐T) network constructed by Cytoscape 3.6.1.

### 2.5 Molecular docking

Based on the C‐T network analysis, the interactions between the ligands and candidate targets were further forecasted with a molecular docking assay. The 3D structures of DA, IG and CE were constructed and stored as Protein Data Bank (PDB) files. The structure information for human Hsp90α protein (1.7 Å, HSP90AA1) was acquired from the PDB (https://www.rcsb.org/). Auto Dock Tools 1.5.6, with the genetic algorithm via default parameters, was used in the docking run, and each compound was docked into the macromolecular target (Zhang et al., 2021). After docking, the best binding modes (protein‐ligand complexes) were obtained, and the results were analyzed and visualized with PyMol software.

### 2.6 Protective effect on SH‐SY5Y neuronal cells

The SH‐SY5Y cell line possesses many characteristics of dopaminergic neurons and is an ideal cell model for PD research (Alrashidi et al., 2021). Cells grown in DMEM were maintained at 37°C in a humidified atmosphere with 5% CO_2_. The effects of DA, IG and CE on SH‐SY5Y cell proliferation were detected using a CCK8 assay (Dojindo, Japan) to evaluate the neuroprotective activity. The experiments were performed as follows: SH‐SY5Y cells (1 × 10^4^ cells per well) were seeded in 96‐well plates and treated with DA, IG and CE (0.5, 1, 2.5, 5, 10, 25, 50 and 100 μM) for 24 h; SH‐SY5Y cells (1 × 10^4^ cells per well) were seeded in 96‐well plates and treated with 3.5 μM MPP for 24 h and subsequently incubated with DA (0.4, 0.5 and 1 μM), IG (0.5, 1 and 2.5 μM), CE (0.5, 1 and 2.5 μM) and curcumin (10 μM) for 24 h. Cell viability was expressed as the equation: cell viability (%) = OD_treated_/OD_control_ × 100%.

### 2.7 Western blot analysis

Western blot was performed as previously described with minor modifications (Zhang et al., 2019). Briefly, harvested cells were lysed in RIPA buffer, and protein concentrations were determined via BCA assay follow the manufacturer's instructions. Total protein (50 μg) was resolved (SDS‐PAGE, 8%–15% gradient gels) and transferred to PVDF membranes. Immunoblotting was carried out using primary antibodies and horseradish peroxidase‐conjugated secondary antibodies. The blots were visualized using a Tanon 5200 automatic chemiluminescence image analysis system (Tanon, Shanghai, China).

The following antibodies were used: anti‐ERK (1:1000, cat no. 4695S, CST), anti‐p‐ERK (1:500, cat no. 9101S, CST), anti‐PI3K (1:1000, cat no. 4249S, CST), anti‐p‐PI3K (1:500, cat no. 4228S, CST), anti‐GAPDH (1:2000, cat no. UM4002, UtiBody), HRP‐conjugated goat anti‐rabbit IgG (1:3000, cat no. bs‐0295G‐HRP, Bioss) and HRP‐conjugated goat anti‐mouse IgG (1:3000, cat no. bs‐0296G‐HRP, Bioss).

### 2.8 Statistical analysis

Data are presented as the mean ± standard deviation (SD) of at least three independent experiments. Statistical significance was analyzed by ANOVA (OmicShare Tools, https://www.omicshare.com/tools) and expressed as *p < .05 and **p < .01.

## 3 RESULTS AND DISCUSSION

### 3.1 Effect of *C. officinalis* on the loss of dopaminergic neurons

**FIGURE 1:**
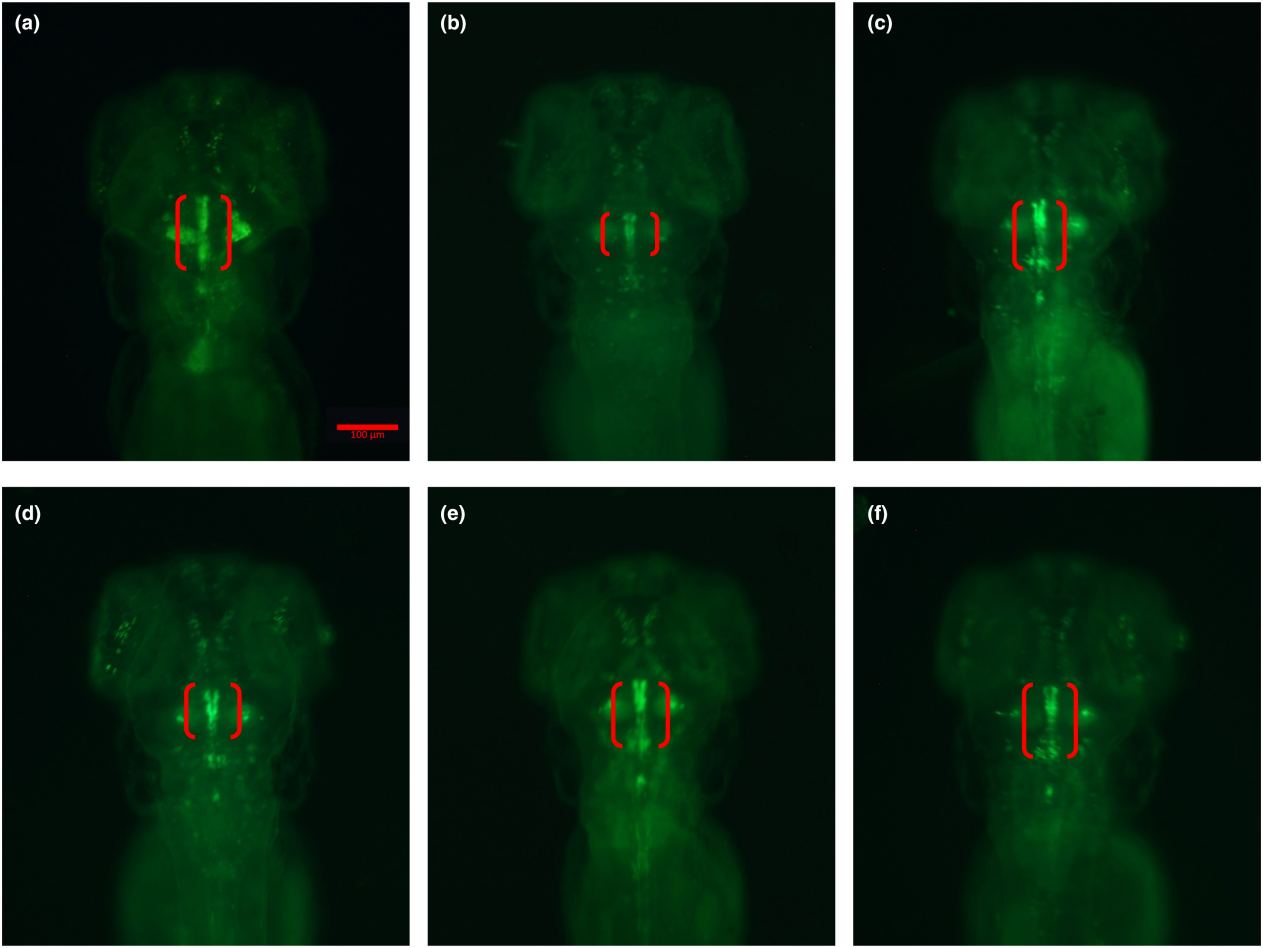
Effect of *C. officinalis* extracts on the loss of DA neurons in the MPTP‐induced zebrafish model. (a) Control group, (b) MPTP group, (c) Positive group, (d) MPTP + 2.5 μg/mL extract, (e) MPTP + 5 μg/mL extract and (f) MPTP + 10 μg/mL extract. DA neurons are indicated by the red brackets; scale bar, 100 μm.

MPTP is commonly used as a neurotoxin that causes selective degeneration of dopaminergic neurons in animal models. The MPTP‐induced zebrafish model has become a well‐accepted technology for evaluating anti‐PD activity (Li et al., 2022). In the Tg (vmat2: GFP) zebrafish line, dopamine neurons were labeled with green fluorescent protein, which enabled region‐specific morphological changes to be visualized under a microscope (Figure 1). The zebrafish larvae of the control group had normally developing dopaminergic neurons in the ventral diencephalon, but the length and fluorescence density of the dopaminergic neuron region decreased after MPTP treatment. *C. officinalis* extracts were shown to restore the defective neurons at concentrations of 2.5–10 μg/mL. In particular, 5–10 and 2.5–10 μg/mL treatments had statistically significant effects on length and fluorescence density, respectively. Relative to the control, 10 μg/mL extract recovered the length and fluorescence density of the dopaminergic neuron region of MPTP‐treated larvae to 96.54% and 87.77%, respectively, which was equivalent to the effect of positive drugs in restoring injured dopamine neurons (Table S1). Hence, the extracts were shown to have powerful potential to alleviate PD symptoms.

### 3.2 Compounds identified in *C. officinalis*

**FIGURE 2:**
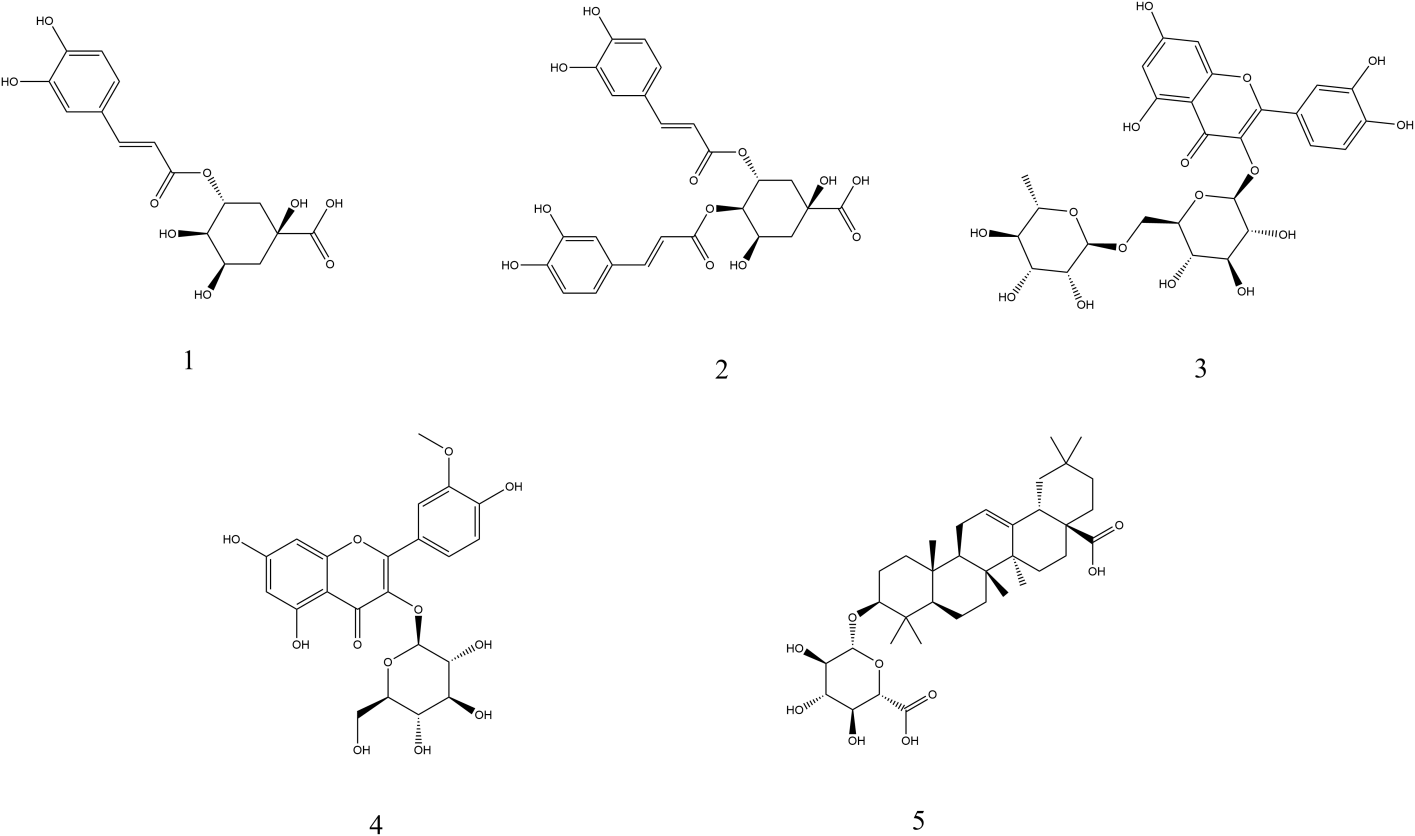
Compounds identified in *C. officinalis* flower extracts using LC‐Q/TOF‐MS/MS. (1) chlorogenic acid, (2) 3,4‐dicaffeoylquinic acid, (3) rutin, (4) isorhamnetin 3‐O‐glucoside and (5) calenduloside E.

Liquid chromatography high‐resolution mass spectrometry was conducted to analyze the *C. officinalis* extract (Figures S1−S6). Chlorogenic acid (1) produced a [M − H]^−^ ion at m/z 353.0871 and a prominent product ion at m/z 191 [quinic acid−H]^−^ (Ye et al., 2010). The 3,4‐dicaffeoylquinic acid (2) produced a [M − H]^−^ ion at m/z 515.1206 and yielded four diagnostic fragment ions at m/z 353 [caffeoylquinic acids−H − H_2_O]^−^, 191 [quinic acid−H]^−^, 179 [caffeoyl−H]^−^ and 173 [quinic acid−H − H_2_O]^−^ (Masike & Madala, 2018). Rutin (3) exhibited a [M − H]^−^ peak at m/z 609.1461 and a major MS/MS fragment at m/z 300 (aglycone) (Jeszka‐Skowron & Zgoła‐Grześkowiak, 2014). Isorhamnetin 3‐O‐glucoside (4) presented a pseudomolecular ion at m/z 477.1040 [M − H]^−^ and released characteristic fragment ions at m/z 314 (aglycone), 285, 271 and 243, which were in good agreement with previously reported data (Schieber et al., 2010). Calenduloside E (5) generated a [M + Cl]^−^ ion at m/z 667.3603 and featured fragment ions at m/z 509 and 455 (oleanolic acid), which have previously been indicated in the literature (Shia et al., 2014). The compounds were confirmed by comparison with reference substances, and the structures are summarized in Figure 2.

### 3.3 Network pharmacology‐based prediction for potential targets

Much previous research in this area has demonstrated chlorogenic acid and rutin to have strong neuroprotective effects (Singh et al., 2020; Singla et al., 2021). In view of the restoring ability of *C. officinalis* extract in dopamine neurons, DA, IG and CE were further investigated for anti‐PD activity using network pharmacology technology. After removing redundant entries, 253 protein targets of the compounds and 130 protein targets of PD, respectively, were obtained to investigate the protein interactions using the String database. The visualized network system for the compounds‐targets is shown in Figure S7, and “degree” (the number of edges connected to the nodes), referring to the importance of the candidate targets, was served as a screening indicator. Consequently, HSP90AA1 (value: 37), PIK3R1 (value: 37), PIK3CA (value: 33) and MAPK1 (ERK2, value: 43) were shown to be core targets with a high “degree”, and the results indicated their potential roles in anti‐PD responses. Moreover, molecular docking and SH‐SY5Y cell assays were applied to extensively validate the mechanism of activity for each compound.

### 3.4 Compound‐target docking results

**FIGURE 3:**
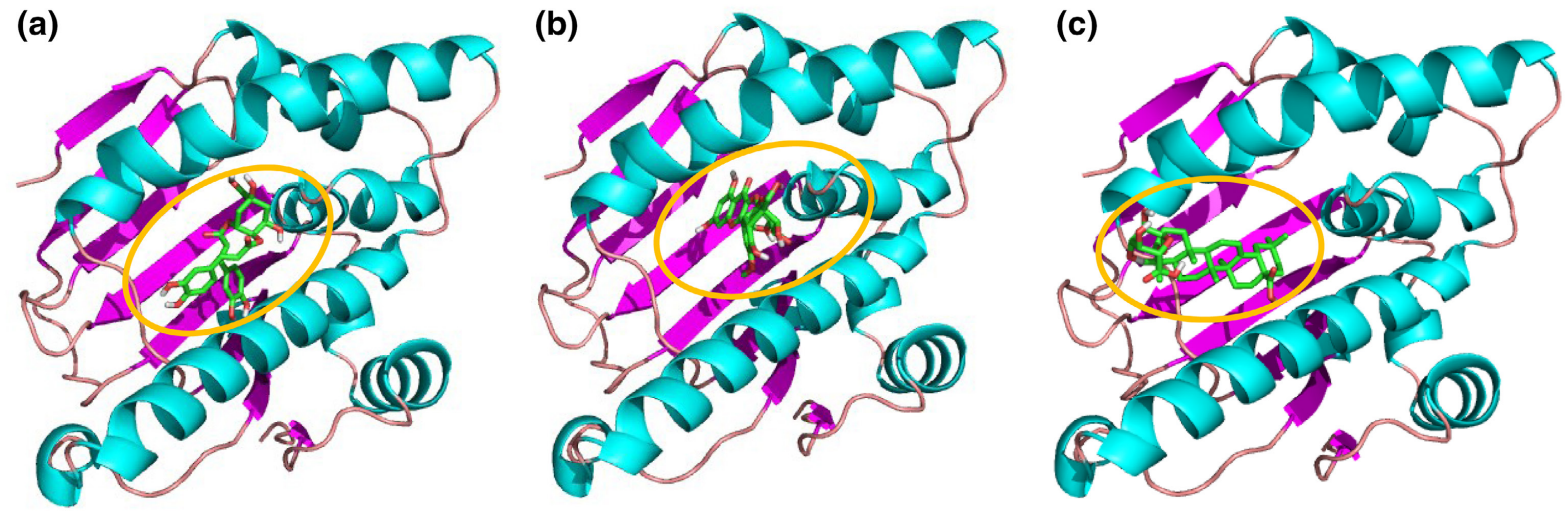
Molecular docking of (a) 3,4‐dicaffeoylquinic acid, (b) isorhamnetin 3‐O‐glucoside and (c) calenduloside E with Hsp90α protein.

As a molecular chaperone, Hsp90α (HSP90AA1) facilitates the correct folding and functionality of its client proteins, as required for cell stabilization and apoptosis regulation (Lyon & Milligan, 2019). In previous work, this has been demonstrated to prevent cultured neurons from necrosis and promote neurite formation, contributing to neuroprotection and repair (Bohush et al., 2019; Dutta et al., 2020; Liao et al., 2021). From our bioinformatics analysis, Hsp90α was predicted to be an effective target of DA, IG and CE. According to the docking results, the three compounds could be docked deep within the pocket region of the Hsp90α protein (Figure 3). The binding energies (∆*G*_b_) of DA and IG were −6.93 kcal/mol and −6.51 kcal/mol, respectively. Binding energy reflects the affinity between ligands and macromolecules, and the results showed that DA and IG expressed strong binding activity to Hsp90α. Six hydrogen bonds were established in the DA‐Hsp90α complex (two with Asn51, two with Gly97, one with Ser52 and one with Phe138); whereas, five hydrogen bonds were generated in the IG‐Hsp90α complex (two with Asn51, one with Lys58, one with Gly135 and one with Tyr139). The binding energy of CE was calculated to be −3.03 kcal/mol, indicating that it bound to Hsp90α with moderate affinity. Moreover, there were no hydrogen bonds formed in the CE‐Hsp90α complex. Other parameters that were revealed after the compounds were docked with Hsp90α are presented in Table S2.

### 3.5 Neuroprotective effect on SH‐SY5Y cells

The chemical toxicity of DA, IG and CE towards SH‐SY5Y cells was determined using the CCK‐8 assay, which indicated the safety of compounds at different concentrations. As shown in Figure S8, the viability of SH‐SY5Y cells was relatively stable under 1 μM of DA. Meanwhile, high viability was exhibited at concentrations of 0.5–2.5 μM in the IG and CE toxicity experiments. Obviously reduced viability at concentrations higher than 10 μM reflected compound‐induced damage to SH‐SY5Y cells. Overall, concentrations of less than 1, 2.5 and 2.5 μM might be regarded as safe limits for DA, IG and CE, respectively.

Subsequently, we examined the effects of DA (0.4–1 μM), IG (0.5–2.5 μM) and CE (0.5–2.5 μM) against MPP‐induced loss of SH‐SY5Y cell viability (Figure S9). Compared with the control group, treatment with MPP for 24 h significantly decreased viability in the SH‐SY5Y cells. According to images obtained using electron microscopy, lesions were characterized by cell shrinkage and pyknosis, or small groups of cells with desquamation. However, cells exposed to the three compounds were able to resume normal growth, and the results showed statistically significant differences (*p* < .01). Our study suggested that SH‐SY5Y cell damage was reversed by DA, IG and CE treatment, indicating their potential protective effects on dopaminergic neurons to alleviate the symptoms caused by PD.

### 3.6 ERK and PI3K in neuroprotection

**FIGURE 4:**
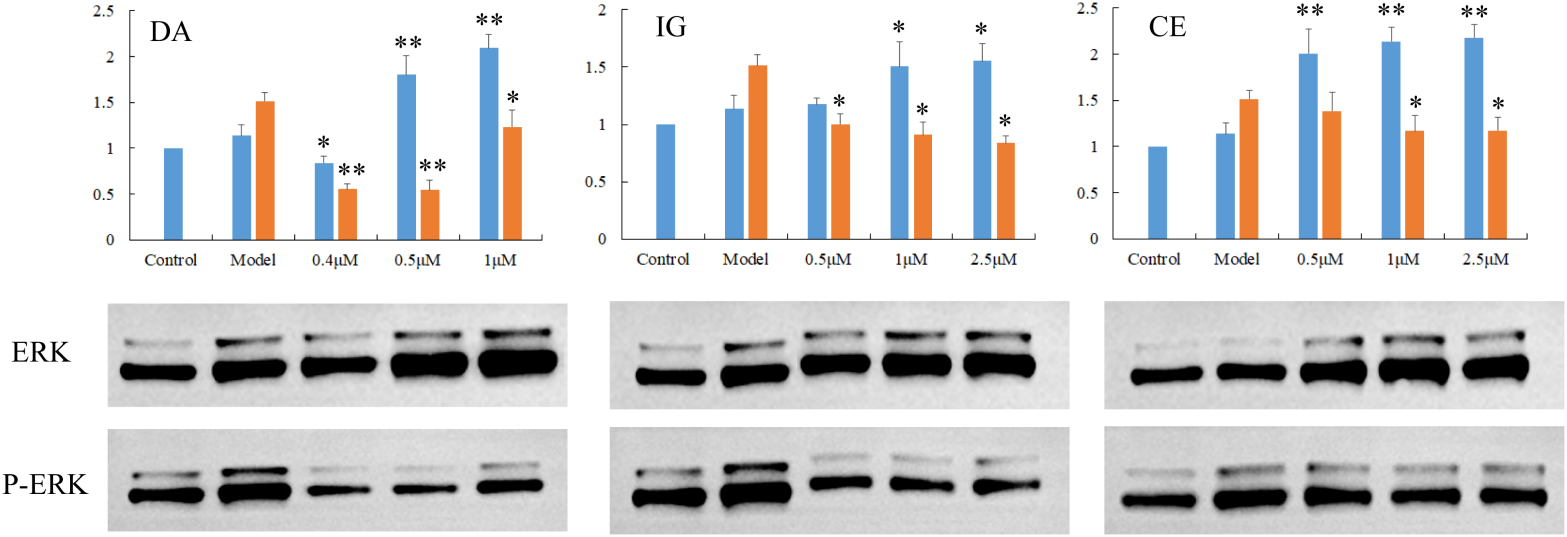
Total and phosphorylated ERK in the SH‐SY5Y cells. Cells without a drug treatment were used as the control group (relative ratio values were set to 1). *p < .05, **p < .01 versus the Model group.

Extracellular regulated kinase (ERK), a critical component of cellular signal transduction pathways, was studied to confirm the role of the tested compounds in anti‐PD neuroprotective activities. Western blotting showed a significant increase in total ERK protein expression at high concentrations of DA, IG and CE. However, ERK phosphorylation and the p‐ERK/ERK ratio were significantly reduced in the intervention groups compared with the models (Figure 4). It has been demonstrated that the toxin increased the p‐ERK/ERK ratio (Amiri et al., 2016), but the activation of ERK was averted by the three compounds used in our experiment, thereby preventing SH‐SY5Y cell death.

**FIGURE 5:**
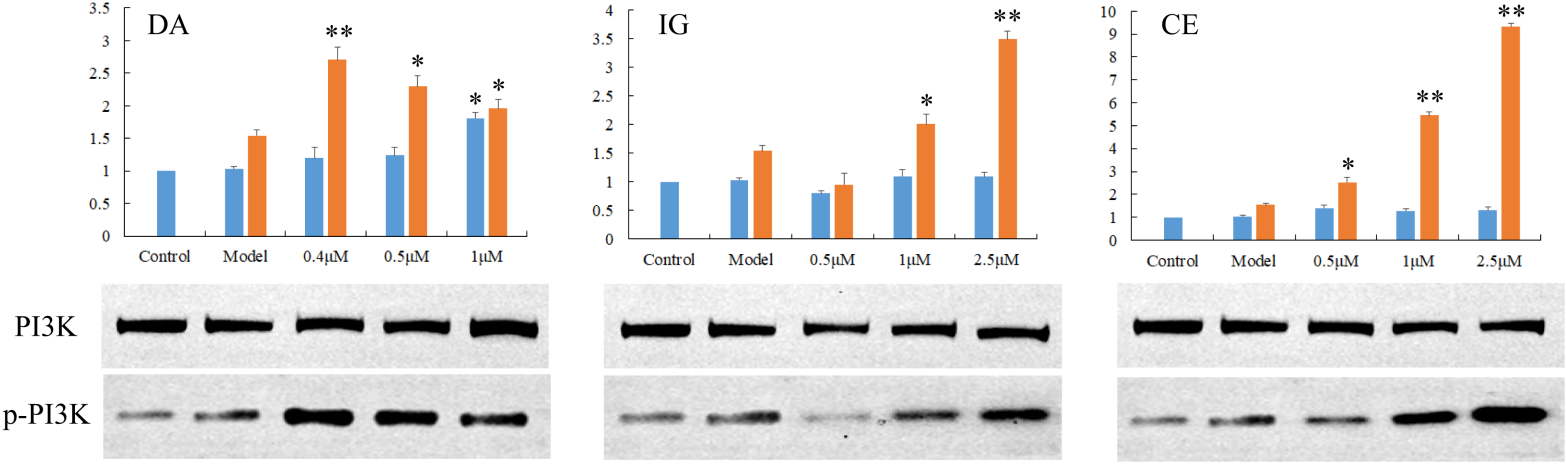
Total and phosphorylated PI3K in the SH‐SY5Y cells. Cells without a drug treatment were used as the control group (relative ratio values were set to 1). *p < .05, **p < .01 versus the Model group.

Phosphoinositide 3‐kinase (PI3K), the two subunits of which are encoded by PIK3CA and PIK3R1 genes, also critically regulated cell survival. Dysregulation in the PI3K/Akt pathway contributed to the loss of dopaminergic neurons in PD. As we show in our results (Figure 5), there were few significant changes in the levels of total PI3K protein in all the experimental groups. Interestingly, both MPP and the compounds presented a tendency to promote the up‐regulation of p‐PI3K, and in particular, a sharp increase was caused at a concentration of 0.4 μM (DA), 2.5 μM (IG) and 2.5 μM (CE), respectively. Previous studies have elucidated that p‐PI3K was up‐regulated in SH‐SY5Y cells treated with toxin (Zhu et al., 2019), and some active ingredients (e.g., flavonoid) helped in protecting dopaminergic neurons by enhancing the phosphorylation of PI3K (Cao et al., 2017; Hu et al., 2018). Consistent with these results, our data revealed activation of the PI3K/AKT signaling pathway in SH‐SY5Y cells via exposure to MPP and the tested compounds. In the toxin group, the anti‐apoptotic PI3K/Akt pathway was actually overactivated. Yalçınkaya et al. (2016) and Dong et al. (2021) illustrated this to presumably be an effort to compensate for increased neuronal death.

Hsp90 has been reported as an appropriate target for modulating the PI3K/Akt and ERK pathways to produce neuroprotective effects. In recent years, many inhibitors were found to block Hsp90 and result in disease‐causing protein degradation, responsible for alleviating neuronal degeneration (Wang et al., 2011; Zhao et al., 2022). Our results provided support for the suggestion that DA, IG and CE bound to Hsp90 and then activated the PI3K/Akt signaling pathway, while concurrently inhibiting the ERK signaling pathway. These events were pivotal to SH‐SY5Y cell survival and may be the underlying mechanisms by which these compounds protect neurons in PD.

## 4 CONCLUSION

In summary, the current study revealed the powerful potential of *C. officinalis* flower extracts to protect against PD symptoms via an in vivo zebrafish assay. Five active compounds were deduced from LC–MS/MS data, including chlorogenic acid, 3,4‐dicaffeoylquinic acid (DA), rutin, isorhamnetin 3‐O‐glucoside (IG) and calenduloside E (CE). DA, IG and CE were further investigated through a combination of network pharmacology, molecular docking and in vitro treatment of SH‐SY5Y cells. Our work has revealed the underlying mechanisms of these compounds in improving the symptoms associated with PD. The compounds were shown to bind to the pocket region of the Hsp90α protein and then concurrently activate the PI3K/Akt signaling pathway and inhibit the ERK signaling pathway. As multifunctional agents capable of regulating these pathways simultaneously, the compounds extracted from *C. officinalis* flowers might have therapeutic significance in the treatment of PD.

## AUTHOR CONTRIBUTIONS

**Xuanming Zhang:** Conceptualization (equal); investigation (equal); supervision (equal); writing – original draft (equal). **Rongchun Wang:** Data curation (equal); validation (equal). **Nataliya Finiuk:** Formal analysis (equal); writing – review and editing (equal). **Rostyslav Stoika:** Formal analysis (equal); writing – review and editing (equal). **Houwen Lin:** Writing – review and editing (equal). **Xue Wang:** Formal analysis (equal); methodology (equal); visualization (equal). **Meng Jin:** Conceptualization (equal); project administration (equal); supervision (equal).

## CONFLICT OF INTEREST STATEMENT

The authors declare that they have no known competing financial interests or personal relationships that could have appeared to influence the work reported in this paper.

## Supporting information


Data S1

## Data Availability

The data that support the findings of this study are available on request from the corresponding author.

## References

[fsn33792-bib-0001] Ak, G. , Zengin, G. , Ceylan, R. , Mahomoodally, M. F. , Jugreet, S. , Mollica, A. , & Stefanucci, A. (2021). Chemical composition and biological activities of essential oils from *Calendula officinalis* L. flowers and leaves. Flavour and Fragrance Journal, 36, 554–563. 10.1002/ffj.3661

[fsn33792-bib-0002] Alrashidi, H. , Eaton, S. , & Heales, S. (2021). Biochemical characterization of proliferative and differentiated SH‐SY5Y cell line as a model for Parkinson's disease. Neurochemistry International, 145, 105009. 10.1016/j.neuint.2021.105009 33684546

[fsn33792-bib-0003] Amiri, E. , Ghasemi, R. , & Moosavi, M. (2016). Agmatine protects against 6‐OHDA‐induced apoptosis, and ERK and Akt/GSK disruption in SH‐SY5Y cells. Cellular and Molecular Neurobiology, 36, 829–838. 10.1007/s10571-015-0266-7 26346882 PMC11482516

[fsn33792-bib-0004] Balestrino, R. , & Schapira, A. H. V. (2020). Parkinson disease. European Journal of Neurology, 27, 27–42. 10.1111/ene.14108 31631455

[fsn33792-bib-0005] Bloem, B. R. , Okun, M. S. , & Klein, C. (2021). Parkinson's disease. The Lancet, 397, 2284–2303. 10.1016/S0140-6736(21)00218-X 33848468

[fsn33792-bib-0006] Bohush, A. , Bieganowski, P. , & Filipek, A. (2019). Hsp90 and its co‐chaperones in neurodegenerative diseases. International Journal of Molecular Sciences, 20, 4976. 10.3390/ijms20204976 31600883 PMC6834326

[fsn33792-bib-0007] Cao, Q. , Qin, L. Y. , Huang, F. , Wang, X. S. , Yang, L. , Shi, H. L. , Wu, H. , Zhang, B. B. , Chen, Z. Y. , & Wu, X. J. (2017). Amentoflavone protects dopaminergic neurons in MPTP‐induced Parkinson's disease model mice through PI3K/Akt and ERK signaling pathways. Toxicology and Applied Pharmacology, 319, 80–90. 10.1016/j.taap.2017.01.019 28185818

[fsn33792-bib-0008] Cruceriu, D. , Diaconeasa, Z. , Socaci, S. , Socaciu, C. , Rakosy‐Tican, E. , & Balacescu, O. (2020). Biochemical profile, selective cytotoxicity and molecular effects of *Calendula officinalis* extracts on breast cancer cell lines. Notulae Botanicae Horti Agrobotanici Cluj‐Napoca, 48, 24–39. 10.15835/nbha4811178

[fsn33792-bib-0009] Della Valle, A. , Dimmito, M. P. , Zengin, G. , Pieretti, S. , Mollica, A. , Locatelli, M. , Cichelli, A. , Novellino, E. , Ak, G. , Yerlikaya, S. , Baloglu, M. C. , Altunoglu, Y. C. , & Stefanucci, A. (2020). Exploring the nutraceutical potential of dried pepper *Capsicum annuum* L. on market from Altino in Abruzzo region. Antioxidants, 9, 400. 10.3390/antiox9050400 32397242 PMC7278808

[fsn33792-bib-0010] Dhingra, G. , Dhakad, P. , & Tanwar, S. (2022). Review on phytochemical constituents and pharmacological activities of plant *Calendula officinalis* Linn. Biological Sciences, 2, 216–228. 10.55006/biolsciences.2022.2205

[fsn33792-bib-0011] Dong, W. W. , Luo, B. , Qiu, C. , Jiang, X. , Shen, B. , Zhang, L. , Liu, W. G. , & Zhang, W. B. (2021). TRIM3 attenuates apoptosis in Parkinson's disease via activating PI3K/AKT signal pathway. Aging, 13, 735–749. 10.18632/aging.202181 PMC7835008 33253119

[fsn33792-bib-0012] Dutta, D. J. , Hashimoto‐Torii, K. , & Torii, M. (2020). Role of heat shock factor 1 in neural development and disorders. In A. A. A. Asea , & P. Kaur (Eds.), Heat shock proteins in inflammatory diseases (Vol. 22, pp. 213–240). Springer International Publishing. 10.1007/7515_2020_10

[fsn33792-bib-0013] Funayama, M. , Nishioka, K. , Li, Y. Z. , & Hattori, N. (2023). Molecular genetics of Parkinson's disease: Contributions and global trends. Journal of Human Genetics, 68, 125–130. 10.1038/s10038-022-01058-5 35821405 PMC9968657

[fsn33792-bib-0014] Hu, M. , Li, F. , & Wang, W. (2018). Vitexin protects dopaminergic neurons in MPTP‐induced Parkinson's disease through PI3K/Akt signaling pathway. Drug Design, Development and Therapy, 12, 565–573. 10.2147/DDDT.S156920 29588573 PMC5859909

[fsn33792-bib-0015] Jeszka‐Skowron, M. , & Zgoła‐Grześkowiak, A. (2014). Analysis of antioxidant activity, chlorogenic acid, and rutin content of *Camellia sinensis* infusions using response surface methodology optimization. Food Analytical Methods, 7, 2033–2041. 10.1007/s12161-014-9847-1

[fsn33792-bib-0016] Li, X. Z. , Gao, D. L. , Paudel, Y. N. , Li, X. , Zheng, M. Z. , Liu, G. P. , Ma, Y. R. , Chu, L. , He, F. T. , & Jin, M. (2022). Anti‐Parkinson's disease activity of *Sanghuangprous vaninii* extracts in the MPTP‐induced zebrafish model. ACS Chemical Neuroscience, 13, 330–339. 10.1021/acschemneuro.1c00656 35044760

[fsn33792-bib-0017] Liao, L. S. , Lu, S. , Yan, W. T. , Wang, S. C. , Guo, L. M. , Yang, Y. D. , Huang, K. , Hu, X. M. , Zhang, Q. , Yan, J. , & Xiong, K. (2021). The role of HSP90α in methamphetamine/hyperthermia‐induced necroptosis in rat striatal neurons. Frontiers in Pharmacology, 12, 716394. 10.3389/fphar.2021.716394 34349659 PMC8326403

[fsn33792-bib-0018] Lyon, M. S. , & Milligan, C. (2019). Extracellular heat shock proteins in neurodegenerative diseases: New perspectives. Neuroscience Letters, 711, 134462. 10.1016/j.neulet.2019.134462 31476356

[fsn33792-bib-0019] Masike, K. , & Madala, N. (2018). Synchronized survey scan approach allows for efficient discrimination of isomeric and isobaric compounds during LC‐MS/MS analyses. Journal of Analytical Methods in Chemistry, 2018, 1–8. 10.1155/2018/2046709 PMC5901820 29805830

[fsn33792-bib-0020] Mohammed, A. B. A. , Yagi, S. , Tzanova, T. , Schohn, H. , Abdelgadir, H. , Stefanucci, A. , Mollica, A. , Mahomoodally, M. F. , Adlan, T. A. , & Zengin, G. (2020). Chemical profile, antiproliferative, antioxidant and enzyme inhibition activities of *Ocimum basilicum* L. and *Pulicaria undulata* (L.) CA Mey. Grown in Sudan. South African Journal of Botany, 132, 403–409. 10.1016/j.sajb.2020.06.006

[fsn33792-bib-0021] Patil, K. , Sanjay, C. J. , Doggalli, N. , Renuka Devi, K. R. , & Harshitha, N. (2022). A review of *Calendula officinalis*‐magic in science. Journal of Clinical and Diagnostic Research, 16, ZE23–ZE27. 10.7860/JCDR/2022/52195.16024

[fsn33792-bib-0022] Schieber, A. , Keller, P. , Streker, P. , Klaiber, I. , & Carle, R. (2010). Detection of isorhamnetin glycosides in extracts of apples (*Malus domestica* cv. "Brettacher") by HPLC‐PDA and HPLC‐APCI‐MS/MS. Phytochemical Analysis, 13, 87–94. 10.1002/pca.630 12018028

[fsn33792-bib-0023] Shia, M. Y. , Yang, Y. , Sun, Y. T. , Cheng, L. M. , Zhao, S. , Xu, H. B. , Fawcett, J. P. , Sun, X. B. , & Gu, J. K. (2014). Pharmacokinetic study of calenduloside E and its active metabolite oleanolic acid in beagle dog using liquid chromatography‐tandem mass spectrometry. Journal of Chromatography B, 951–952, 129–134. 10.1016/j.jchromb.2014.01.036 24556278

[fsn33792-bib-0024] Singh, S. S. , Rai, S. N. , Birla, H. , Zahra, W. , Rathore, A. S. , Dilnashin, H. , Singh, R. , & Singh, S. P. (2020). Neuroprotective effect of chlorogenic acid on mitochondrial dysfunction‐mediated apoptotic death of DA neurons in a parkinsonian mouse model. Oxidative Medicine and Cellular Longevity, 2020, 6571484. 10.1155/2020/6571484 32566093 PMC7273475

[fsn33792-bib-0025] Singla, R. K. , Agarwal, T. , He, X. F. , & Shen, B. R. (2021). Herbal resources to combat a progressive & degenerative nervous system disorder‐Parkinson's disease. Current Drug Targets, 22, 609–630. 10.2174/1389450121999201013155202 33050857

[fsn33792-bib-0026] Tanideh, N. , Ghafari, V. , Ebrahimi, R. , Habibagahi, R. , Koohi‐Hosseinabadi, O. , & Iraji, A. (2020). Effects of *Calendula officinalis* and *Hypericum perforatum* on antioxidant, anti‐inflammatory, and histopathology indices of induced periodontitis in male rats. Journal of Dentistry, 21, 314–321. 10.30476/dentjods.2020.83660.1056 33344682 PMC7737925

[fsn33792-bib-0027] Trager, M. H. , Wilkins, K. B. , Koop, M. M. , & Bronte‐Stewart, H. (2020). A validated measure of rigidity in Parkinson's disease using alternating finger tapping on an engineered keyboard. Parkinsonism & Related Disorders, 81, 161–164. 10.1016/j.parkreldis.2020.10.047 33157435 PMC7770028

[fsn33792-bib-0028] Tung, Y. T. , Wu, M. F. , Lee, M. C. , Wu, J. H. , Huang, C. C. , & Huang, W. C. (2019). Antifatigue activity and exercise performance of phenolic‐rich extracts from *Calendula officinalis*, *Ribes nigrum*, and *Vaccinium myrtillus* . Nutrients, 11, 1715. 10.3390/nu11081715 31349650 PMC6722806

[fsn33792-bib-0029] Vitale, S. , Colanero, S. , Placidi, M. , Emidio, G. D. , Tatone, C. , Amicarelli, F. , & D'Alessandro, A. M. (2022). Phytochemistry and biological activity of medicinal plants in wound healing: An overview of current research. Molecules, 27, 3566. 10.3390/molecules27113566 35684503 PMC9182061

[fsn33792-bib-0030] Wang, G. H. , Krishnamurthy, K. , & Tangpisuthipongsa, D. (2011). Protection of murine neural progenitor cells by the Hsp90 inhibitor 17‐allylamino‐17‐demethoxygeldanamycin in the low nanomolar concentration range. Journal of Neurochemistry, 117, 703–711. 10.1111/j.1471-4159.2011.07239.x 21395580

[fsn33792-bib-0031] Yalçınkaya, N. , Haytural, H. , Bilgiç, B. , Özdemir, Ö. , Hanağası, H. , Küçükali, C. İ. , Özbek, Z. , Akcan, U. , İdrisoğlu, H. A. , Gürvit, H. , & Tüzün, E. (2016). Expression changes of genes associated with apoptosis and survival processes in Parkinson's disease. Neuroscience Letters, 615, 72–77. 10.1016/j.neulet.2016.01.029 26806867

[fsn33792-bib-0032] Yang, Y. Y. , Qian, J. C. , Mei, J. M. , Zhong, K. , & Niu, C. S. (2020). In vivo detection of metabolic changes in the striatum of proteasomal inhibition‐induced Parkinson's disease in rats using proton MR spectroscopy at 9.4 T. The International Journal of Neuroscience, 130, 153–160. 10.1080/00207454.2019.1667783 31516042

[fsn33792-bib-0033] Ye, J. X. , Wei, W. , Quan, L. H. , Liu, C. Y. , Chang, Q. , & Liao, Y. H. (2010). An LC–MS/MS method for the simultaneous determination of chlorogenic acid, forsythiaside A and baicalin in rat plasma and its application to pharmacokinetic study of Shuang‐huang‐lian in rats. Journal of Pharmaceutical and Biomedical Analysis, 52, 625–630. 10.1016/j.jpba.2010.01.035 20153596

[fsn33792-bib-0034] Zhang, X. M. , Han, L. W. , Li, P. H. , Zhang, S. S. , Zhang, M. Q. , Li, X. B. , Chu, J. , Wang, L. Z. , Tu, P. F. , Zhang, Y. , & Liu, K. C. (2021). Region‐specific biomarkers and their mechanisms in the treatment of lung adenocarcinoma: A study of *Panax quinquefolius* from Wendeng, China. Molecules, 26, 6829. 10.3390/molecules26226829 34833921 PMC8623508

[fsn33792-bib-0035] Zhang, X. M. , Shi, Y. P. , Wang, L. Z. , Li, X. B. , Zhang, S. S. , Wang, X. M. , Jin, M. , Hsiao, C. D. , Lin, H. W. , Han, L. W. , & Liu, K. C. (2019). Metabolomics for biomarker discovery in fermented black garlic and potential bioprotective responses against cardiovascular diseases. Journal of Agricultural and Food Chemistry, 67, 12191–12198. 10.1021/acs.jafc.9b04073 31588747

[fsn33792-bib-0036] Zhao, L. , Ding, L. D. , Xia, Z. H. , Sheng, P. , Shen, M. M. , Cai, Z. M. , & Yan, B. C. (2022). A network‐based approach to investigate the neuroprotective effects and mechanisms of action of Huangqi‐Chuanxiong and Sanleng‐Ezhu herb pairs in the treatment of cerebral ischemic stroke. Frontiers in Pharmacology, 13, 844186. 10.3389/fphar.2022.844186 35401166 PMC8984614

[fsn33792-bib-0037] Zhu, J. G. , Dou, S. S. , Jiang, Y. L. , Bai, B. , Chen, J. , Wang, C. M. , & Cheng, B. H. (2019). Apelin‐36 exerts the cytoprotective effect against MPP+‐induced cytotoxicity in SH‐SY5Y cells through PI3K/Akt/mTOR autophagy pathway. Life Sciences, 224, 95–108. 10.1016/j.lfs.2019.03.047 30905782

