# Supplementary material for: Active compounds from Calendula officinalis flowers act via PI3K and ERK signaling pathways to offer neuroprotective effects against Parkinson's disease
Source: Food Sci Nutr. 2023 Oct 26;12(1):450–8. doi: 10.1002/fsn3.3792 (PMC10804118; doi:10.1002/fsn3.3792)
Supplement: Supplementary file 1 — Data S1 [file FSN3-12-450-s001.docx]

Supporting information

**Active compounds from *Calendula officinalis* flowers act via PI3K and ERK signaling pathways to offer neuroprotective effects against Parkinson’s disease**

Xuanming Zhang ^1^, Rongchun Wang ^1^, Nataliya Finiuk ^2^, Rostyslav Stoika ^2^, Xue Wang ^1^, Houwen Lin ^3^, Meng Jin ^1,^ *

*^1^ Engineering Research Center of Zebrafish Models for Human Diseases and Drug Screening, Biology Institute, Qilu University of Technology (Shandong Academy of Sciences), Jinan 250103, China*

*^2^ Department of Regulation of Cell Proliferation and Apoptosis, Institute of Cell Biology, National Academy of Sciences of Ukraine, Lviv 79005, Ukraine*

*^3^ Research Center for Marine Drugs, State Key Laboratory of Oncogenes and Related Genes, Department of Pharmacy, School of Medicine, Shanghai Jiao Tong University, Shanghai 200127, China*

*Corresponding author: Meng Jin, mjin1985@hotmail.com; Tel: 86-531-82605331.


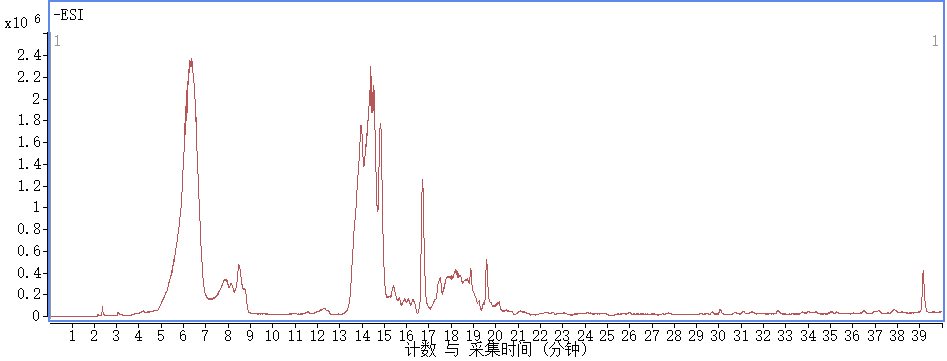


**Fig. S1** LC-MS chromatograms of the five abundant compounds in *C. officinalis* extract.


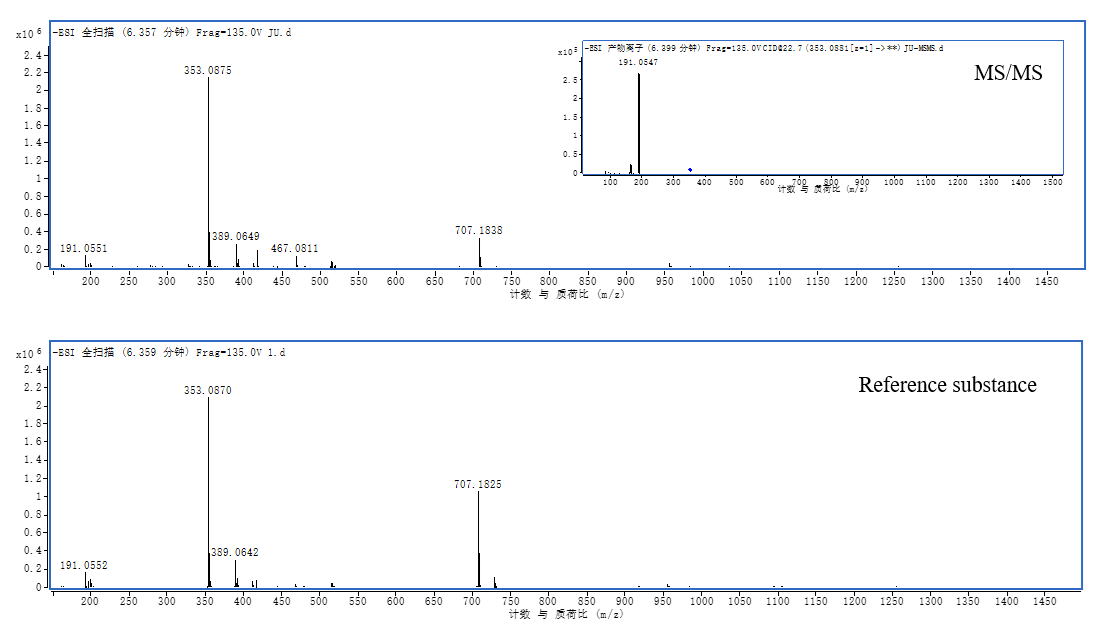


**Fig. S2** MS and MS/MS spectra of chlorogenic acid (**1**) in negative mode.


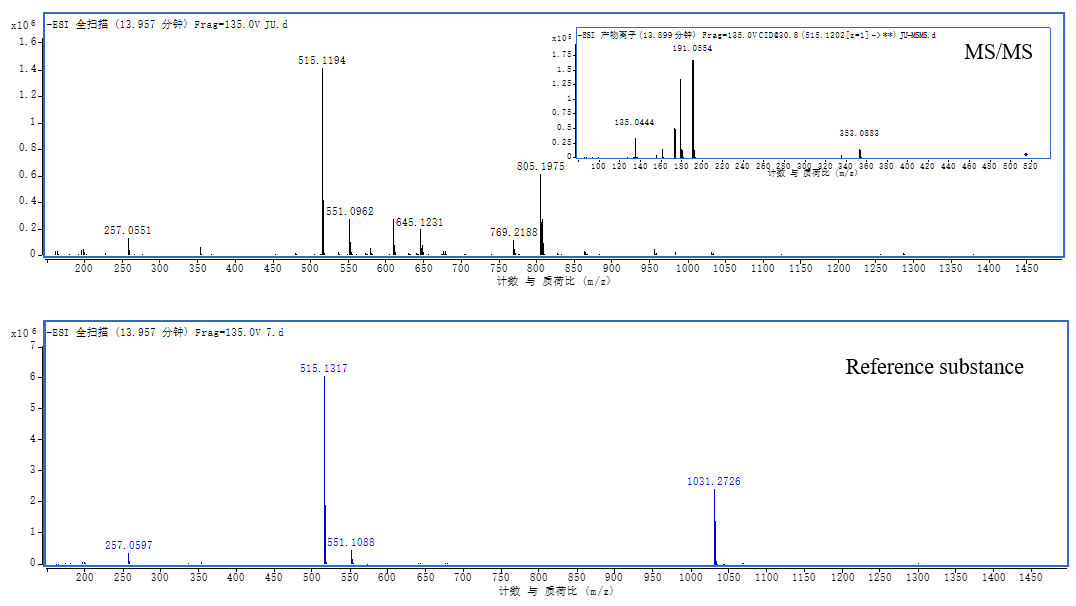


**Fig. S3** MS and MS/MS spectra of 3,4-dicaffeoylquinic acid (**2**) in negative mode.


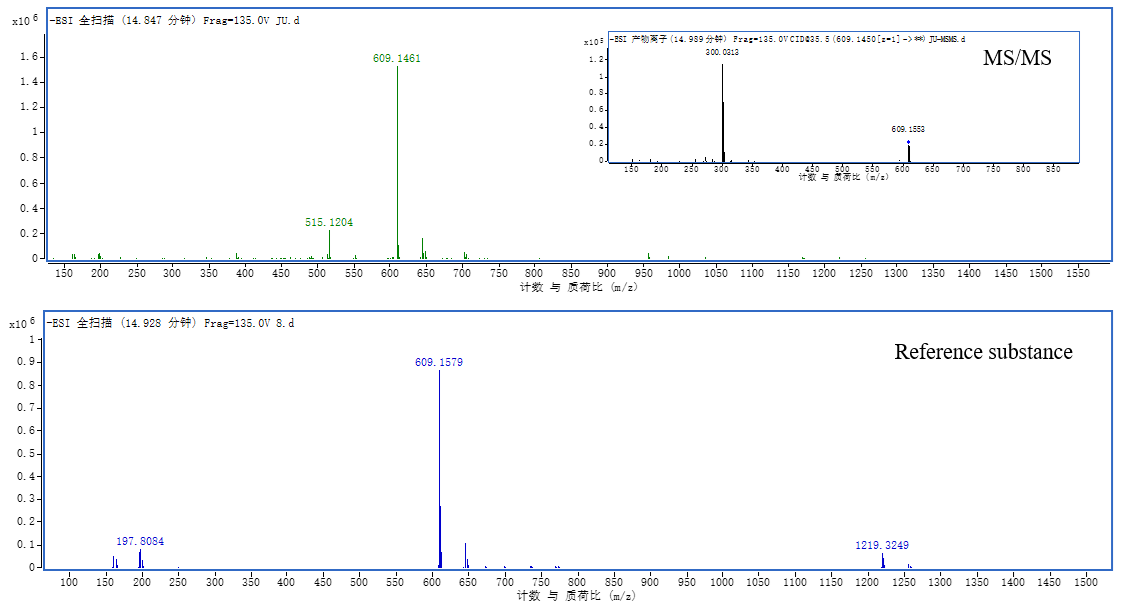


**Fig. S4** MS and MS/MS spectra of rutin (**3**) in negative mode.


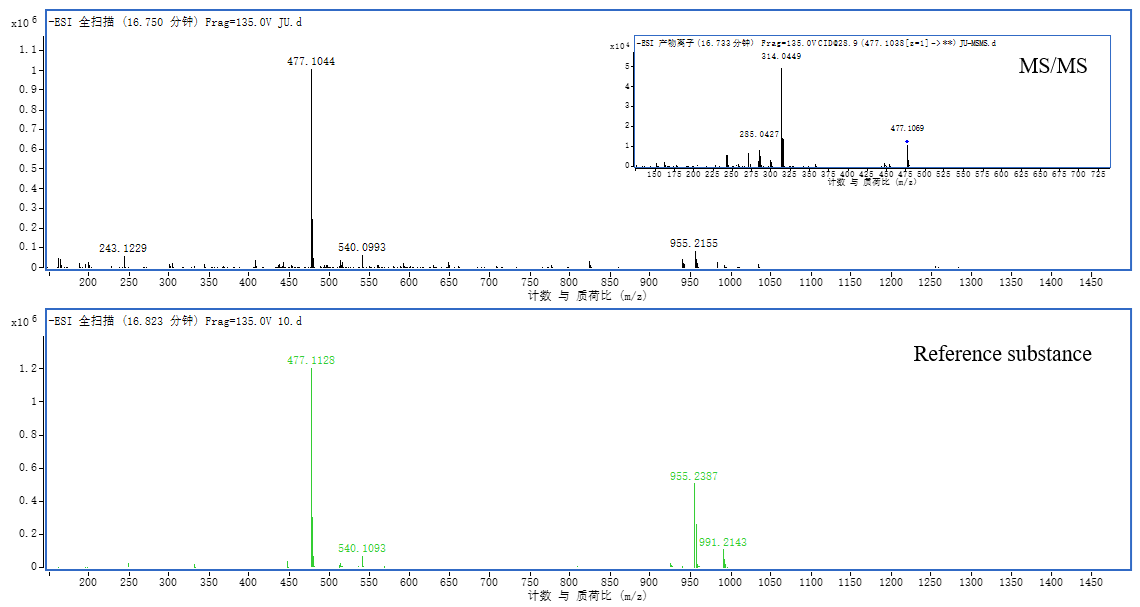


**Fig. S5** MS and MS/MS spectra of isorhamnetin 3-O-glucoside (**4**) in negative mode.


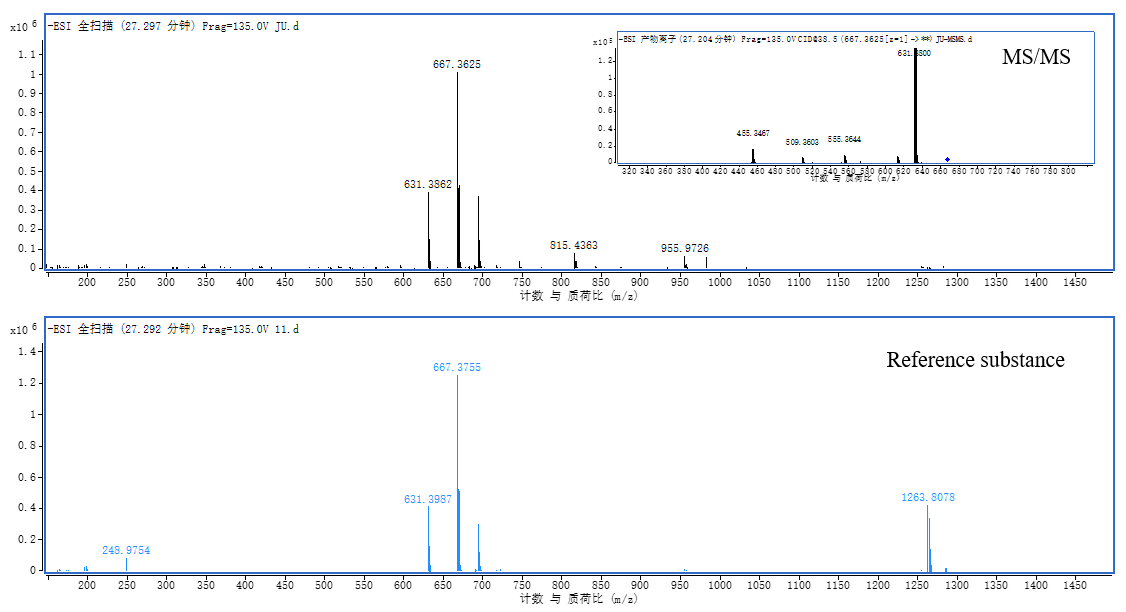


**Fig. S6** MS and MS/MS spectra of calenduloside E (**5**) in negative mode.


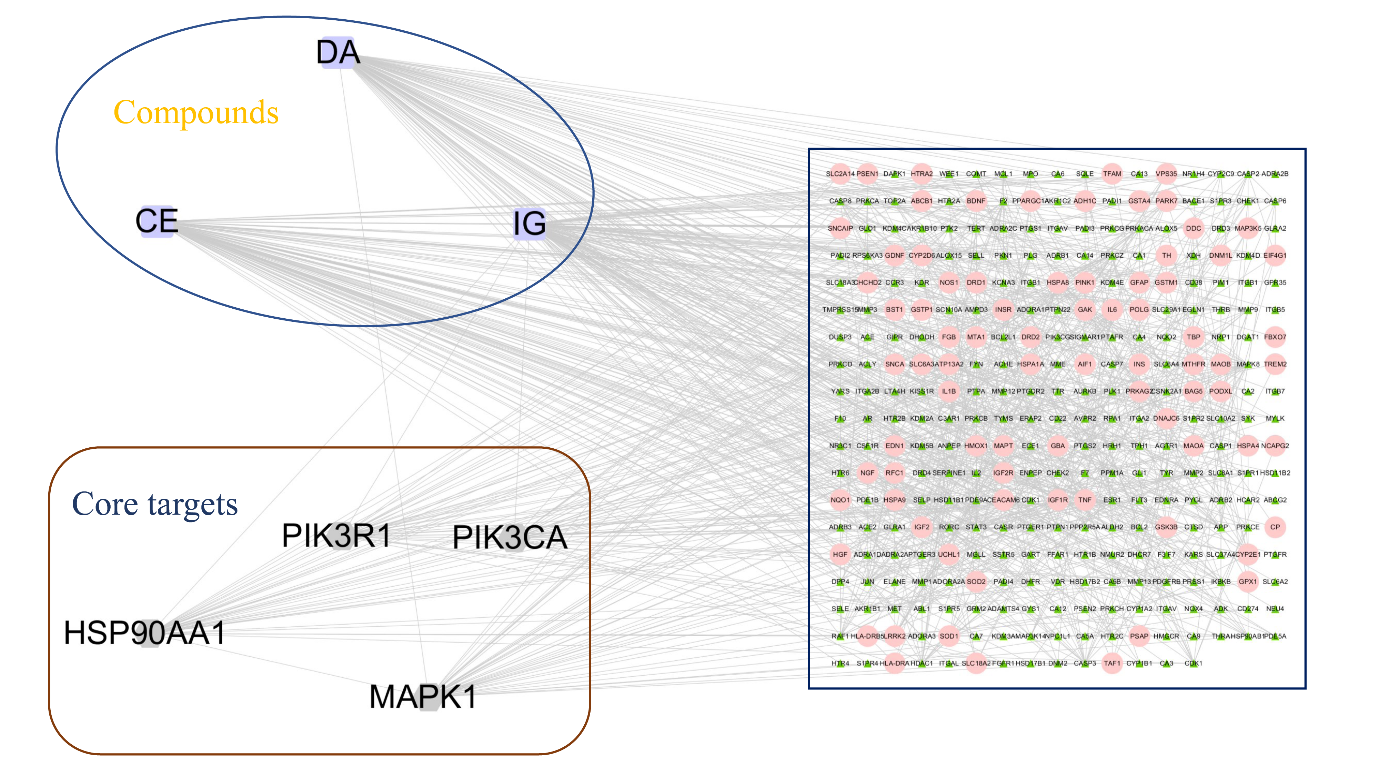


**Fig. S7** A candidate-target network for DA, IG and CE activity against PD. The compounds and their core targets are indicated individually; the circular nodes and triangle nodes represent other predicted targets and therapeutic targets, respectively.


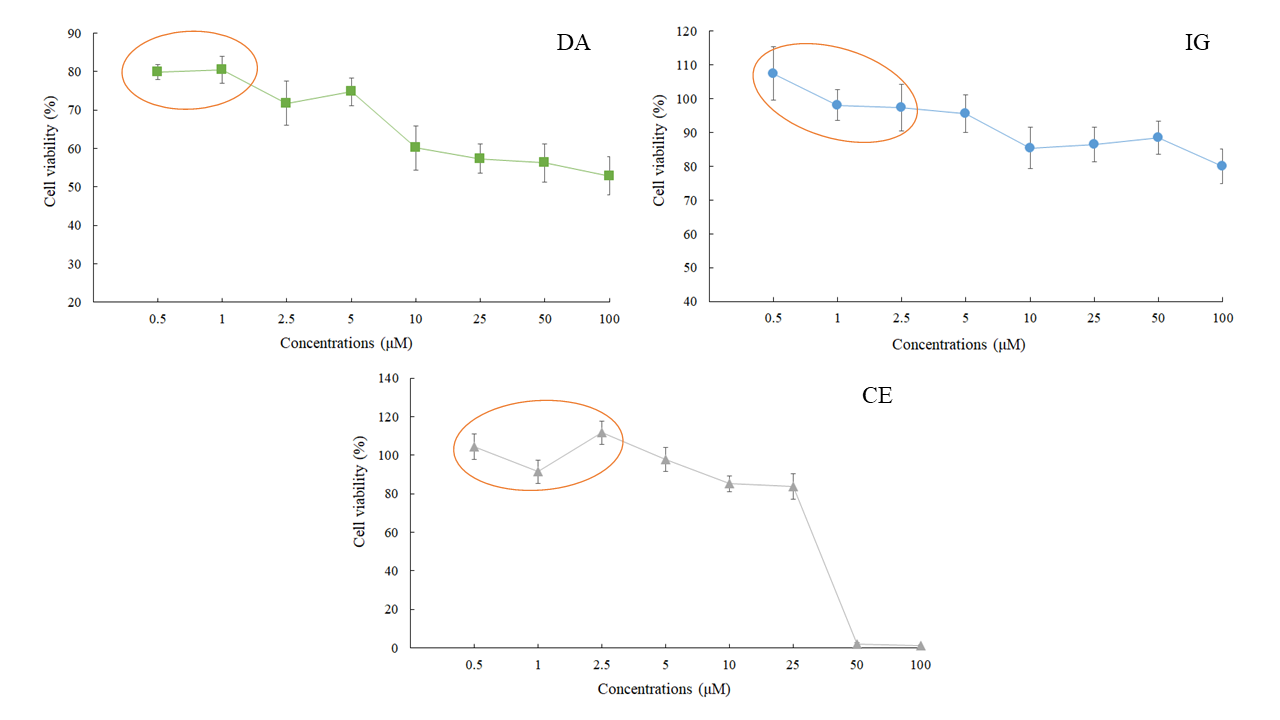


**Fig. S8** Chemical toxicity of DA, IG and CE towards SH-SY5Y cells.


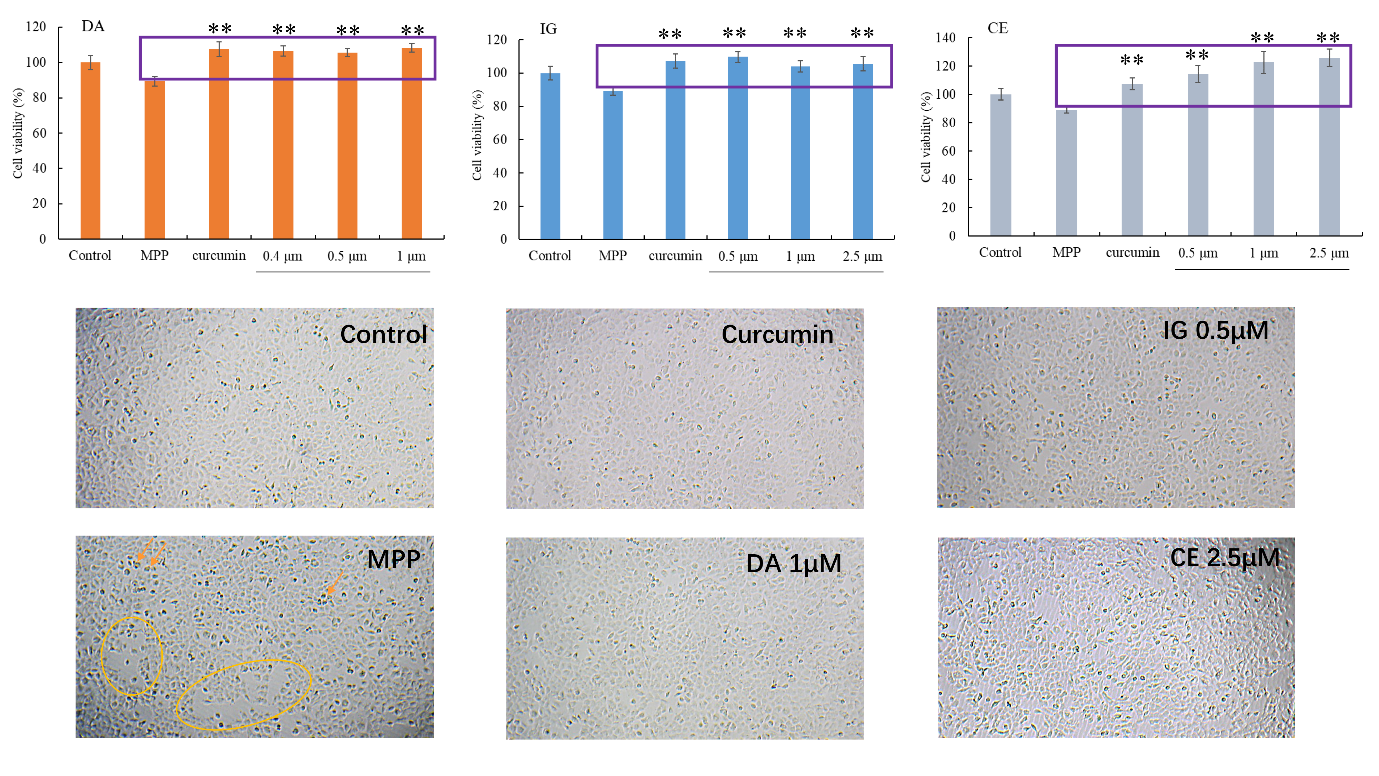


**Fig S9** Effects of DA, IG and CE in protecting SH-SY5Y cell viability from toxin-induced injury. Representative microscopy-based images of SH-SY5Y cells are presented (** *p* < 0.01 vs. the MPP group).

**Table S1** The length and fluorescence density of DA neuron region in Zebrafish (% of Control)

| Group | Dose | Length (%) | Fluorescence (%) |
| --- | --- | --- | --- |
| Control | — | — | — |
| MPTP | 50 μM | 54.73 ± 3.27 | 68.42 ± 4.62 |
| MPTP + nomifensine | 50 μM +30 μM | 96.98 ± 2.29** | 84.81 ± 6.15* |
| MPTP + extract | 50 μM + 2.5 μg/mL | 60.19 ± 1.69 | 83.46 ± 1.4* |
| MPTP + extract | 50 μM + 5 μg/mL | 88.06 ± 0.39** | 83.82 ± 3.9* |
| MPTP + extract | 50 μM + 10 μg/mL | 96.54 ± 1.65** | 87.77 ± 1.85** |

**p* < 0.05 and ***p* < 0.01 vs. the MPTP group

**Table S2**. The results of binding energy calculation

| Compound | Binding energy ∆ *G*_b_ (kcal/mol) | Intermolecular energy  (kcal/mol) | Electrostatic energy (kcal/mol) | Unbound energy  (kcal/mol) |
| --- | --- | --- | --- | --- |
| DA | −6.93 | −11.7 | −0.12 | −6.28 |
| IG | −6.51 | −9.2 | −0.46 | −4.55 |
| CE | −3.03 | −5.71 | −0.24 | −4.33 |
